# Cerebrospinal fluid–related tissue damage in multiple sclerosis patients with iron rim lesions

**DOI:** 10.1177/13524585231155639

**Published:** 2023-02-19

**Authors:** Matthias Wittayer, Claudia E Weber, Maximilian Kittel, Michael Platten, Lucas Schirmer, Hayrettin Tumani, Achim Gass, Philipp Eisele

**Affiliations:** Department of Neurology, Mannheim Center of Translational Neurosciences (MCTN), Medical Faculty Mannheim, Heidelberg University, Mannheim, Germany; Department of Neurology, Mannheim Center of Translational Neurosciences (MCTN), Medical Faculty Mannheim, Heidelberg University, Mannheim, Germany; Institute for Clinical Chemistry, Medical Faculty Mannheim, Heidelberg University, Mannheim, Germany; Department of Neurology, Mannheim Center of Translational Neurosciences (MCTN), Medical Faculty Mannheim, Heidelberg University, Mannheim, Germany/German; Consortium of Translational Cancer Research (DKTK), Clinical Cooperation Unit Neuroimmunology and Brain Tumor Immunology, German Cancer Research Center (DKFZ), Heidelberg, Germany; Department of Neurology, Mannheim Center of Translational Neurosciences (MCTN), Medical Faculty Mannheim, Heidelberg University, Mannheim, Germany/Mannheim Institute for Innate Immunoscience, Medical Faculty Mannheim, Heidelberg University, Mannheim, Germany; Department of Neurology, Ulm University, Ulm, Germany; Department of Neurology, Mannheim Center of Translational Neurosciences (MCTN), Medical Faculty Mannheim, Heidelberg University, Mannheim, Germany; Department of Neurology, Mannheim Center of Translational Neurosciences (MCTN), Medical Faculty Mannheim, Heidelberg University, Mannheim, Germany

**Keywords:** Multiple sclerosis, MRI, iron rim lesions, chronic active lesions, CSF

## Abstract

**Background:**

In multiple sclerosis (MS), iron rim lesions (IRLs) are associated with pronounced tissue damage, higher disease severity and have been suggested as an imaging marker of chronic active inflammation behind the blood–brain barrier indicating progression. Furthermore, chronic intrathecal compartmentalized inflammation has been suggested to be a mediator of a cerebrospinal fluid (CSF)–related tissue damage.

**Objective:**

To investigate CSF markers of intrathecal inflammation in patients with at least one IRL compared to patients without IRLs and to investigate tissue damage in lesions and normal-appearing white matter (NAWM) with proximity to CSF spaces.

**Methods:**

A total of 102 patients (51 with at least 1 IRL and 51 age-/sex-matched patients without IRL) scanned with the same 3T magnetic resonance imaging (MRI) and having CSF analysis data were included.

**Results:**

Patients with at least one IRL had higher disability scores, higher lesion volumes, lower brain volumes and a higher intrathecal immunoglobulin G (IgG) synthesis. Apparent diffusion coefficient (ADC) values in IRLs were higher compared to non-IRLs. We observed a negative linear correlation of ADC values in all tissue classes and distance to CSF, which was stronger in patients with high IgG quotients.

**Conclusion:**

IRLs are associated with higher intrathecal IgG synthesis. CSF-mediated intrathecal smouldering inflammation could explain a CSF-related gradient of tissue damage.

## Introduction

In multiple sclerosis (MS), chronic compartmentalized inflammation represents a fundamental driver of clinical progression.^1^ Compartmentalized inflammation proceeds behind a nearly closed blood–brain barrier in the brain parenchyma as well as in the meninges, where lymphoid follicle-like structures of predominantly B-cells and plasma cells activate mainly microglial cells in the cortex via soluble substances like interferon gamma (INF-γ) and tumour necrosis factor (TNF)-alpha, leading to pronounced mainly subpial cortical demyelination.^2^ Furthermore, the permanent low-burning inflammation is presented by chronic active MS lesions, which are characterized by a peripheral rim of iron-laden proinflammatory microglia/macrophages associated with ongoing slow degree of demyelination and axonal loss, thus reflecting a compartmentalized inflammation.^3,4^

In vivo, chronic active lesions can be identified on iron-sensitive magnetic resonance imaging (MRI) sequences as paramagnetic or iron rim lesions (PRLs or IRLs, respectively).^4,5^ Previous studies demonstrated that the presence of IRLs is associated with more severe clinical disability, progressive disease course and central nervous system atrophy.^5–8^ Furthermore, there is increasing evidence that chronic, intrathecal compartmentalized inflammation represents an important mediator of a cerebrospinal fluid (CSF)–related tissue damage.^9,10^ Indeed, previous studies have demonstrated a CSF-related gradient with a more severe tissue matrix damage in the normal-appearing white matter (NAWM) and chronic lesions in proximity to the inner and outer surfaces of the brain.^11–16^

Against this background, the aims of this study were (a) to evaluate CSF markers of intrathecal inflammation in MS patients with at least one IRL compared to patients without IRLs and (b) to investigate their relationship with tissue damage in lesions (both IRLs and non-IRLs) and the NAWM, as well as with distance to the adjacent CSF spaces.

## Material and methods

### Patients

We retrospectively screened our database to identify two groups of patients (MS patients with at least one IRL on susceptibility-weighted imaging (SWI) and an age- and sex-matched group without IRLs) who met the following inclusion criteria: (1) diagnosis of definite MS according to the 2010 criteria,^17^ (2) at least 18 years of age, (3) MRI acquired on the same 3T MRI system using identical imaging parameters and (4) availability of CSF-related data (obtained as part of diagnostic purposes). In cases CSF was obtained more than once, the data closest to the time of MRI were used for further analysis as suggested previously.^18,19^ Signs of acute inflammatory activity (relapses and/or presence of acute contrast-enhancing lesions) at the time point of lumbar puncture (LP) were manually recorded from medical and radiology reports. Exclusion criteria were the presence of neurological diseases other than MS, cardiovascular or respiratory disease and missing or insufficient MRI data. On the days of MRI examinations, age, sex, disease duration, disease phenotype, disease-modifying therapies (DMTs), Expanded Disability Status Scale (EDSS) including the pyramidal functional system score, relapses and number of contrast-enhancing lesions were assessed.

### MRI

MRI was performed on a 3T MR system (MAG-NETOM Skyra, Siemens Healthineers (Erlangen, Germany), 20-channel head coil) including the following sequences: three-dimensional (3D) magnetization-prepared rapid acquisition gradient-echo (MPRAGE; echo time (TE) = 2.49 ms, repetition time (TR) = 1900 ms, inversion time (TI) = 900 ms, field-of-view (FOV) = 240 mm, spatial resolution = 0.9 × 0.9 × 0.9 mm); 3D fluid-attenuated inversion recovery (FLAIR; TE = 398 ms, TR = 5000 ms, TI = 1800 ms, FOV = 240 mm, resolution = 0.5 × 0.5 × 0.9 mm); diffusion-weighted echo planar images (DWIs; TE = 68 ms, TR = 5300 ms, *b* = 0/1000 s/mm^2^, FOV = 220 mm, slice thickness 4 mm, resolution = 0.98 × 0.98 × 4.0 mm) including apparent diffusion coefficient (ADC) calculations; SWI (TE = 20 ms, TR = 27 ms, FOV = 220 mm, slice thickness (ST) = 1.5 mm, voxel-size = 0.9 × 0.9 × 1.5 mm (until September 2018 acquired after contrast-injection as a delay before acquisition of post-contrast T1-weighted images, afterwards prior contrast agent administration) and T1-weighted images (TE = 2.5 ms, TR = 225 ms, FOV = 220 mm) acquired 10 minutes after contrast-injection (single dose gadoterate meglumine). SWIs were generated automatically by the scanner software provided by the manufacturer.

### CSF analysis

All CSF analyses were performed at the Institute for Clinical Chemistry, Medical Faculty Mannheim, Heidelberg University, including the following parameters: leukocyte count, CSF protein concentration, CSF glucose and lactate, CSF and serum of albumin, immunoglobulin G (IgG), immunoglobulin M (IgM) and immunoglobulin A (IgA) including the corresponding ratios (*Q*_alb_, *Q*_IgG_, *Q*_IgM_, *Q*_IgA_), percentage and absolute values of the intrathecally synthesized immunoglobulins and oligoclonal bands. Blood–CSF barrier dysfunction (*Q*_alb_) and intrathecal Ig synthesis were determined based on the method of Reiber.^20^ Since the upper limit of the reference range for an intact blood–CSF barrier function is age-dependent, pathological cutoffs for *Q*_alb_ were defined by an age-adjusted equation as described previously.^21^

### MRI post-processing analysis

In all patients, two readers (with 7 and 15 years’ experience, respectively), blinded to clinical and CSF data, evaluated MR images jointly. If uncertainty remained regarding potential lesion classification, an additional reviewer (with 30 years’ experience) was consulted for final determination of lesion classification. SWIs were evaluated for the presence of chronic lesions with a characteristic hypointense rim as described previously.^22^

Brain volumes, normalized for subject head size, were calculated on lesion-filled 3D MPRAGE data sets using SIENAX (https://fsl.fmrib.ox.ac.uk/fsl/fslwiki/SIENA), automatic segmentation of deep grey matter (DGM) volumes was performed with FSL FIRST (https://fsl.fmrib.ox.ac.uk/fsl/fslwiki/FIRST).

Using SPM12 (Version 7771, Functional Imaging Laboratory, Wellcome Centre for Human Neuro-imaging, London, UK; https://www.fil.ion.ucl.ac.ukon), 3D MPRAGE and 3D-FLAIR data sets were co-registered to the quantitative ADC maps applying a rigid body transformation. Lesion segmentation was performed semi-automatically on 3D FLAIR images using the lesion prediction algorithm of the Lesion Segmentation Toolbox (https://www.statistical-modelling.de/lst.html). An experienced reader manually corrected lesion masks using MRIcroGL (https://nitrc.org/projects/mricrogl). According to their appearance on SWI, an IRL- and a non-IRL-mask was generated. To investigate voxel-wise CSF-related tissue damage in lesions and the NAWM, tissue segmentation was achieved using the segmentation algorithm of SPM12 on MPRAGE images. Lesion masks were subtracted from the white matter masks to produce an NAWM mask. The CSF tissue mask was then binarized using FSL (https://fsl.fmrib.ox.ac.uk/fsl/fslwiki/FSL) with a probability threshold of 0.95. We used a conservative threshold of 0.95 since lower thresholds tend to overestimate CSF spaces. The binary CSF mask was then overlaid with a distance transformation map created with Convert 3D software (http://www.itksnap.org/pmwiki/pmwiki.php?n=Convert3D.Doc2). To account for partial volume effects, the first 2 mm around the borders of the CSF space were excluded from further analysis. Furthermore, we excluded all lesion voxels <1.5 mm. Non-zero voxels were converted to ASCII values, resulting in a two-dimensional (2D) matrix of distance and ADC values for each voxel in the lesion and NAWM mask.

To investigate the association between magnitude of intrathecal Inflammation (IgG synthesis) and a CSF-related gradient of tissue damage, the whole study cohort was dichotomized according to the median IgG quotient (MS patients with ‘high’ IgG quotient and MS patients with ‘low’ IgG quotient). Voxel-wise analysis of tissue damage in this supplementary analysis was performed analogously to the analysis already described above.

### Statistical analysis

Statistical analyses were conducted using R (version 4.0.2) and IBM SPSS Statistics (version 27, Chicago, IL, USA). Correlational analyses of imaging data were conducted using the Spearman rank correlations. Descriptive and exploratory analyses, as well as confirmatory multivariate models, were conducted using the appropriate tests depending on the distribution of the variable data. Normality was confirmed or rejected by calculation of Kolmogorov–Smirnov test and inspection of the histograms and Q–Q plots. As most of our data was non-normally distributed, non-parametric statistics were used. In the description of the analyses, we chose to report medians and interquartile range (IQR) accordingly.

### Standard protocols and procedures

The local ethics committee approved this study. Patient consent was waived by our institutional review board due to the retrospective nature of the study and the lack of patient interaction.

## Results

### Clinical and volumetric MRI assessment

Characteristics of the study population according to clinical and radiological data are summarized in Table 1. A total of 102 MS patients were included in the final analysis. These included 51 MS patients with at least 1 IRL on SWI and 51 age- and sex-matched MS patients without IRLs.

**Table 1.** Clinical and MRI characteristics of the study population.

	MS patients with ⩾1 IRL	MS patients without IRLs	Statistics
Number of patients	51	51	
Age, years, median (IQR)	40 (29–47)	40 (29–47)	Mann–Whitney *U* test, *p* = 0.989
Sex, *n* (female/male)	33/18	33/18	χ^2^ test, *p* = 1.0
RRMS/SPMS/PPMS, *n*	46/4/1	49/2/0	χ^2^ test, *p* = 0.414
DMT, *n* (%)	37 (72.5%)	43 (84.3%)	χ^2^ test, *p* = 0.149
High efficacy DMT, *n* (%)	15 (29.4%)	15 (25.3%)	χ^2^ test, *p* = 0.657
Disease duration, years (IQR)	2 (1–8)	3 (1–7)	Mann–Whitney *U* test, *p* = 0.865
EDSS, median (range)	2.5 (0–7.5)	1.75 (0–6)	Mann–Whitney *U* test, *p* = 0.001
Pyramidal functional system score, median (range)	1 (0–4)	0.5 (0–3)	Mann–Whitney *U* test, *p* = 0.004
Number of IRLs, median (IQR)	1 (1–2)	–	
Number of CE lesions, median (IQR)	0 (0–0)	0 (0–0)	Mann–Whitney *U* test, *p* = 0.852
Patients with relapses, *n* (%)	3 (0.6%)	4 (0.8%)	χ^2^ test, *p* = 0.695
Grey matter volume (mL), median (IQR)	769.35 (721.03–792.85)	788.74 (761.55–824.58)	Mann–Whitney *U* test, *p* = 0.002
White matter volume (mL), median (IQR)	725.72 (689.16–755.33)	749.64 (713.22–787.58)	Mann–Whitney *U* test, *p* = 0.007
Deep grey matter volume (mL), median (IQR)	33.67 (31.4–36.99)	36.01 (34.56–37.94)	Mann–Whitney *U* test, *p* = 0.001
IRL volume (mL), median (IQR)	0.61 (0.32–1.36)	–	
Non-IRL volume (mL), median (IQR)	4.85 (1.79–11.31)	2.39 (0.44–4.47)	Mann–Whitney *U* test, *p* = 0.004
Total lesion volume (mL), median (IQR)	4.85 (2.6–12.45)	2.39 (0.44–4.47)	Mann–Whitney *U* test, *p* < 0.001

MRI: magnetic resonance imaging; MS: multiple sclerosis; IRL: iron rim lesion; IQR: interquartile range; RRMS: relapsing-remitting multiple sclerosis; SPMS: secondary progressive multiple sclerosis; PPMS: primary progressive multiple sclerosis; DMT: disease-modifying therapy; EDSS: Expanded Disability Status Scale; CE: contrast-enhancing.

MS patients with at least one IRL had significantly higher disability scores (both EDSS and pyramidal functional system), a significantly higher T2 lesion volume and significantly lower brain volumes compared to patients without IRLs. We found no statistically significant differences between both groups regarding DMT, high efficacy DMT, disease duration, relapses and number of contrast-enhancing lesions.

### CSF analysis

**Figure 1.**
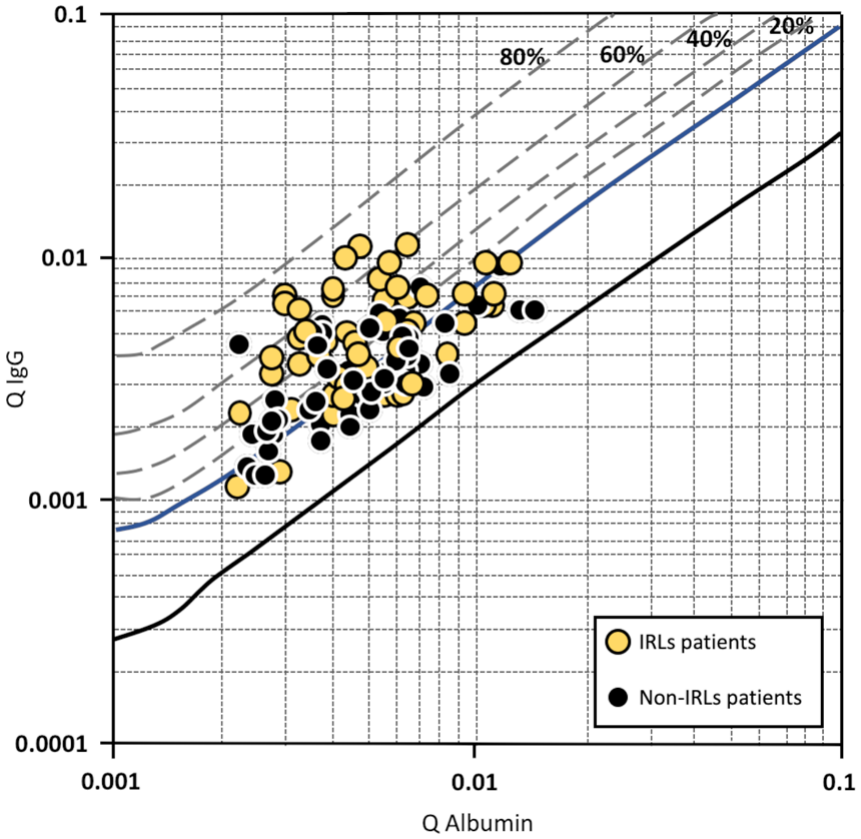
Reibergram of patients with at least one iron rim lesion (IRL) and patients without IRLs. The four dashed lines allow the fraction (20, 40, 60 and 80%) of intrathecal immunoglobulin (Ig) synthesis relative to the total immunoglobulin content present in CSF. The upper reference range limit for the CSF/serum albumin quotient is age-dependent and therefore not marked in the figure.

The results of the CSF analysis are reported in Table 2. Compared to patients without IRLs, significantly more patients with at least one IRL on MRI demonstrated an intrathecal IgG synthesis. Furthermore, patients with IRLs had a significantly higher IgG quotient determined by Reibergram (see also Figure 1) and a significantly higher percentage and absolute values of intrathecal IgG synthesis, while we did not find statistically significant differences between both groups regarding intrathecal IgA or IgM synthesis.

**Table 2.** Cerebrospinal fluid characteristics of the study population.

	MS patients with ⩾1 IRL	MS patients without IRLs	Statistics
Time between lumbar puncture and MRI, years, median (IQR)	1 (0–6)	3 (1–6)	Mann–Whitney *U* test, *p* = 0.433
Number of patients with relapses at time point of lumbar puncture, *n* (%)	30 (58.8%)	39 (76.5%)	χ^2^ test, *p* = 0.057
Number of patients with contrast-enhancing lesions at time point of lumbar puncture, *n* (%)	27 (52.9%)	35 (68.6%)	χ^2^ test, *p* = 0.105
CSF leukocyte count (cells/µL), median (IQR)	5 (1–15)	2 (1–5)	Mann–Whitney *U* test, *p* = 0.202
CSF total protein (mg/L), median (IQR)	419.1 (333.6–537.25)	365.5 (280.8–452.6)	Mann–Whitney *U* test, *p* = 0.026
CSF glucoses (mg/dL), median (IQR)	62 (58–70)	60 (54–66)	Mann–Whitney *U* test, *p* = 0.065
CSF lactate (mM), median (IQR)	1.8 (1.62–2.16)	1.75 (1.53–2.1)	Mann–Whitney *U* test, *p* = 0.261
CSF albumin (mg/L), median (IQR)	201.5 (156.25–286.75)	224 (141.5–274.5)	Mann–Whitney *U* test, *p* = 0.441
Serum albumin (g/L), median (IQR)	42.6 (40.23–46.3)	42 (40–44.6)	Mann–Whitney *U* test, *p* = 0.354
Quotient albumin CSF/serum, median (IQR)	4.81 (3.55–6.53)	5.23 (3.48–6.48)	Mann–Whitney *U* test, *p* = 0.755
Blood–CSF barrier dysfunction, *n* (%)	13 (26%)	12 (23.5%)	χ^2^ test, *p* = 0.774
Quotient IgG CSF/serum, median (IQR)	5.17 (3.28–7.55)	3.43 (2.43–4.97)	Mann–Whitney *U* test, *p* < 0.001
Intrathecal IgG synthesis, *n* (%)	37 (74%)	28 (54.9%)	χ^2^ test, *p* = 0.045
Percentage intrathecal IgG synthesis, median (IQR)	24.1 (0–57.63)	1.55 (0–6.96)	Mann–Whitney *U* test, *p* = 0.001
Absolute intrathecal IgG synthesis (mg/L), median (IQR)	13.05 (0–29.61)	1.55 (0–6.96)	Mann–Whitney *U* test, *p* = 0.001
Quotient IgA CSF/serum, median (IQR)	1.63 (0.99–2.16)	1.49 (1.15–2.06)	Mann–Whitney *U* test, *p* = 0.938
Intrathecal IgA synthesis, *n* (%)	4 (8%)	5 (10%)	χ^2^ test, *p* = 0.775
Percentage intrathecal IgA synthesis, median (IQR)	0 (0–0)	0 (0–0)	Mann–Whitney *U* test, *p* = 0.744
Absolute intrathecal IgA synthesis (mg/L), median (IQR)	0 (0–0)	0 (0–0)	Mann–Whitney *U* test, *p* = 0.788
Quotient IgM CSF/serum, median (IQR)	0.52 (0.26–1.41)	0.43 (0.26–0.66)	Mann–Whitney *U* test, *p* = 0.231
Intrathecal IgM synthesis, *n* (%)	14 (29%)	14 (28%)	χ^2^ test, *p* = 0.898
Percentage intrathecal IgM synthesis, median (IQR)	0 (0–8.9)	0 (0–7.7)	Mann–Whitney *U* test, *p* = 0.887
Absolute intrathecal IgM synthesis (mg/L), median (IQR)	0 (0–0.15)	0 (0–0.65)	Mann–Whitney *U* test, *p* = 0.682
Oligoclonal bands positive, *n* (%)	47 (92.2%)	42 (82.4%)	χ^2^ test, *p* = 0.138

MS: multiple sclerosis; IRL: iron rim lesion; MRI: magnetic resonance imaging; IQR: interquartile range; CSF: cerebrospinal fluid; IgG: immunoglobulin G; IgM: immunoglobulin M; IgA: immunoglobulin A.

### ADC values in lesions and the NAWM

In patients with at least one IRL on MRI, median ADC values in IRLs were significantly higher compared to non-IRLs (1.09 (IQR) = 1.03–1.24) × 10^−3^ mm^2^/s vs 1.04 (IQR = 0.97–1.13) × 10^−3^ mm^2^/s; *p* = 0.007, Wilcoxon test). Median ADC values in non-IRLs did not statistically differ in both patient groups (1.04 (IQR = 0.97–1.13) × 10^−3^ mm^2^/s vs 1.03 (IQR = 0.97–1.1) × 10^−3^ mm^2^/s; *p* = 0.468, Mann–Whitney *U* test). ADC values in lesions (both IRLs and non-IRLs) were significantly higher compared to the NAWM (Wilcoxon test, *p* < 0.05 for all comparisons), whereas we observed no statistically significant differences in ADC values in the NAWM in patients with at least one IRL (0.77 (IQR = 0.75–0.79) × 10^−3^ mm^2^/s) compared to patients without IRLs (0.76 (IQR = 0.74–0.78) × 10^−3^ mm^2^/s; *p* = 0.088, Mann–Whitney *U* test).

### CSF-related tissue damage in lesions and the NAWM

**Figure 2.**
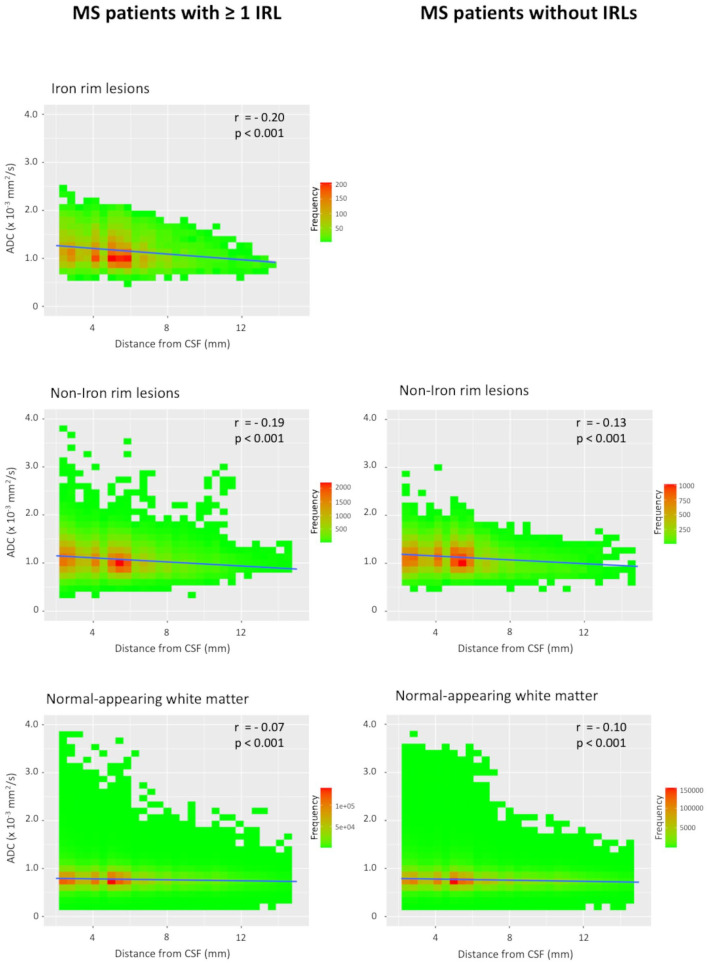
Scatterplots of voxel-wise ADC values in iron rim lesions (IRLs), non-iron rim lesions and the normal-appearing white matter with regard to the distance from the cerebrospinal fluid (CSF) in patients with ⩾1 IRL and patients without IRLs.

**Figure 3.**
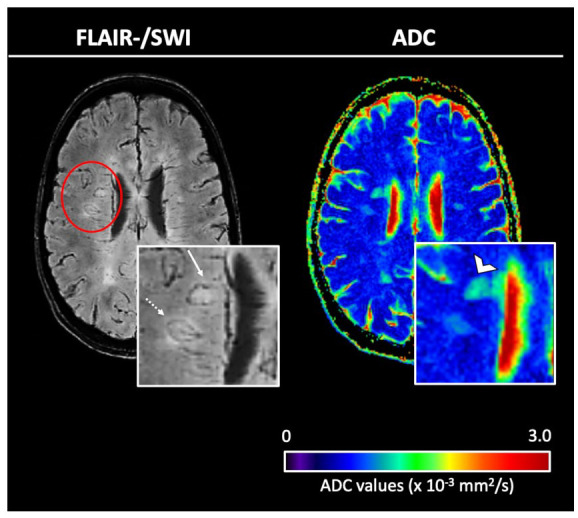
Left: co-registered, overlaid fluid-attenuated inversion recovery (FLAIR)/susceptibility-weighted images (SWI), right: quantitative maps of the apparent diffusion coefficient (ADC). FLAIR/SWI demonstrates representative examples of two iron rim lesions (IRLs); one periventricular IRL (arrow) and one deep white matter IRL (dotted arrow). Note the higher ADC values in the periventricular lesion (open arrowhead) compared to the lesion localized in the deep white matter.

**Figure 4.**
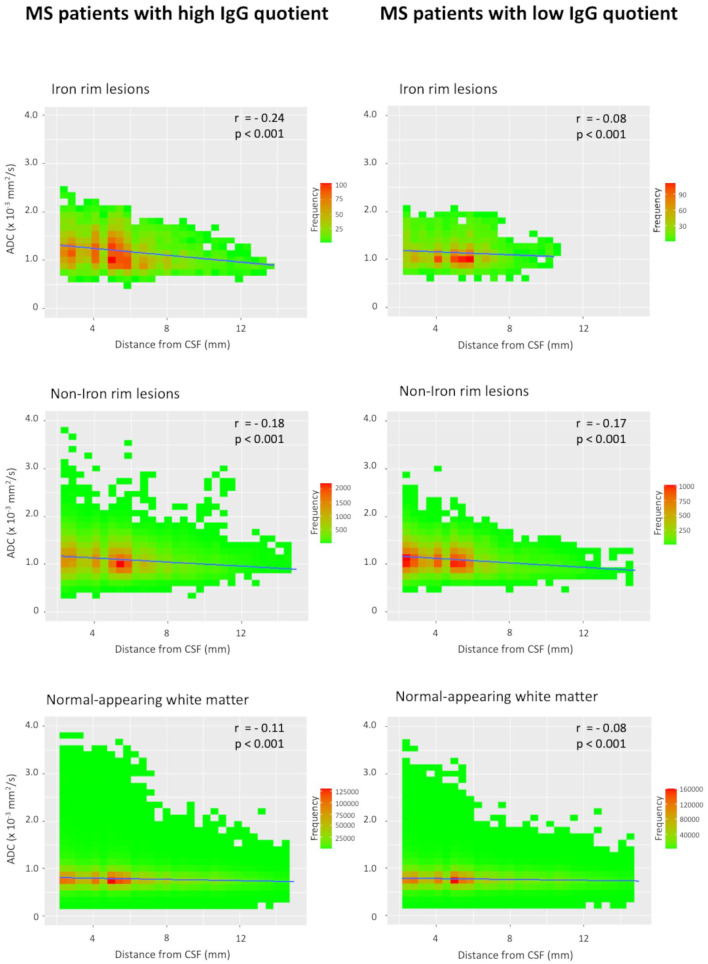
Scatterplots of voxel-wise ADC values in iron rim lesions (IRLs), non-iron rim lesions and the normal-appearing white matter with regard to the distance from the cerebrospinal fluid (CSF) in patients with ‘high’ IgG quotients and patients with ‘low’ IgG quotients.

Voxel-wise ADC values of lesions and the NAWM with regard to the distance from the CSF space in patients with ⩾1 IRL and patients without IRLs are plotted in Figure 2. We observed a linear negative correlation of ADC values in all tissue classes (IRLs, non-IRLs, NAWM) and distance from the CSF space, with higher ADC values closer to the CSF space. A representative example is shown in Figure 3. This negative correlation was stronger in patients with ‘high’ IgG quotients (MS patients with ⩾1 IRL, *n* = 32; MS patients without IRLs, *n* = 19) compared to the ‘low’ IgG group (MS patients with ⩾1 IRL, *n* = 19; MS patients without IRLs, *n* = 32). The corresponding scatterplots are demonstrated in Figure 4. *Z*-matrices for comparison-of-correlations of linear correlations of ADC values and distance from CSF are provided in Supplemental Tables S1 and S2.

## Discussion

In MS, IRLs have been suggested as an imaging marker for chronic active inflammation behind the blood–brain barrier, indicating progression. A recent study also investigated the relationship between IRLs and clinical, MRI and CSF profiles.^18^ Besides an association of IRLs with worse disease severity and lower brain volumes, the authors also found a pathologically elevated albumin quotient as a marker of blood–CSF barrier disruption in MS patients with IRLs.^18^ We now provide some additional observations regarding the association between IRLs, pathogenic CSF profiles and a CSF-related tissue injury.

In this study, MS patients with at least one IRL also showed more aggressive clinical and MRI features, indicating their relevance as a novel imaging marker of disease severity and progression.^5–8^ In addition, IRL patients had significantly higher levels of intrathecal IgG production as represented by Reibergram (IgG quotient), as well as by percentage and absolute values of intrathecally synthesized fractions. On quantitative ADC maps, a sensitive MRI method to quantify the severity of tissue damage in lesions and normal-appearing brain tissue,^23,24^ we found significantly higher ADC values in IRLs compared to non-IRLs, emphasizing their destructive nature.^4–6^ Furthermore, we observed an inversely proportional tissue damage in lesions (both IRLs and non-IRLs) and the NAWM to the distance from the CSF space. This result is in line with previous studies that demonstrated a periventricular-related tissue injury of T2-hyperintense MS lesions.^11–16^ Of note, patients with ‘high’ IgG quotients showed a greater magnitude of negative correlation with ADC values compared to patients with ‘low’ IgG quotients.

One possible hypothesis for this CSF-related tissue injury contains a chronic compartmentalized intrathecal inflammation consisting of B-lymphocyte activation and activated microglia.^9,10^ The results of our CSF analysis support this assumption. Indeed, we found significantly higher levels of intrathecal IgG synthesis indicating a pronounced compartmentalized inflammation in MS patients with at least one IRL compared to patients without IRLs. Interestingly, a previous study demonstrated that MS patients with intrathecal IgG synthesis had a higher risk and shorter time to EDSS worsening during follow-up^19^ and patients with IRLs ultimately showed more severe clinical disability and a progressive disease course.^5–8^ Of note, some studies also reported that intrathecal IgM synthesis is associated with a more severe disease course,^25,26^ whereas in this study, intrathecal IgM synthesis did not statistically differ between both groups. The lack of correlation with IgM could be interpreted to mean that the presence of IRLs is pathoimmunologically mediated by IgG rather than IgM.

Ongoing tissue damage induced by B-lymphocytes, specifically located in the meninges, includes complement-mediated injury and release of immune activating molecules and proinflammatory cytokines.^2^ Furthermore, activated astrocytes and microglia (the latter specifically a characteristic hallmark of IRLs) produce B-cell activating factors, supporting in turn B-cell survival^2^ and thus resulting in a potentially vicious cycle of a self-sustained chronic compartmentalized neuroinflammation. It seems conceivable that, through diffusion into the brain parenchyma, these neurotoxic factors lead to demyelination, axonal degeneration and eventually disability progression.

CSF-mediated damage in proximity to the ventricles might not only spread from ventricular ependyma but also from the venous perivascular ‘Virchow–Robin spaces’, which are connected to the subarachnoid space.^16^ IRLs are mainly located in periventricular regions,^8,22^ where the vein density is the highest.^27^ Interestingly, a previous study demonstrated the iron rim at the lesion edge is characterized by an extensive presence of CD68-positive demyelinating inflammatory infiltrates (macrophages, activated microglia) that distribute along veins crossing the lesion edge.^28^ Furthermore, the authors observed CD8-positive T-lymphocytes in the perivascular space of veins and capillaries,^28^ suggesting that both T- and B-lymphocyte-mediated factors play a deleterious role in tissue damage near the inner surface of the brain. Besides tissue damage observed near the ventricles, previous studies also found significantly lower volumes in the distant cortical grey matter in patients with IRLs.^5,18^ One possible explanation for this would be that tissue damage in periventricular regions is linked to that of the cortex, suggesting a shared mechanism of injury.^12,13^

This study has potential limitations. First, this was a retrospective evaluation of an existing patient population. This is particularly important with regard to the interpretation of the CSF results. However, the average interval between CSF analysis and time point of MRI in this study was similar to that in a previous study.^18^ Patients with acute relapses have higher CSF leukocyte counts than patients with chronic progressive or inactive disease,^29^ and previous studies demonstrated that the CSF leukocyte count and the albumin CSF/serum quotient correlate with proximity of lesions to the lumbar CSF space and the temporal latency of the LP at the time of relapse.^30,31^ While CSF leukocyte counts might decrease under DMTs, the humoral response (IgG synthesis) usually remains unchanged.^32,33^ However, some studies also reported a reduced intrathecal IgG synthesis or a disappearance of oligoclonal IgG bands during DMT.^32^ Furthermore, recent studies suggested that DMTs also might have a beneficial, but delayed effect on tissue damage in IRLs.^34,35^ With regard to the time interval between CSF analysis and MRI assessment, it is also important to mention that previous studies demonstrated that in a small percentage of IRLs, the rim even wanes and finally disappears during follow-up.^4,6,36,37^ Finally, our results are somewhat in contrast to a recent retrospective study in that we found a significantly higher IgG quotient, percentage and absolute intrathecal IgG synthesis in IRL patients, whereas this was not observed by the other group.^18^ Therefore, a prospective study including more patients would be highly interesting.

In conclusion, IRLs are associated with higher intrathecal IgG synthesis and thus both point in the same direction, indicating progression. Whether IRLs can be used as an imaging marker of intrathecal compartmentalized inflammation can only be speculated at present and requires larger studies to further distinguish a temporal from causal relationship. Furthermore, our results suggest that tissue damage in lesions and the NAWM is related to distance to CSF. Whether this CSF-related gradient of tissue damage is related to intrathecal inflammation or other factors needs to be investigated in future studies.

## Supplemental Material

sj-doc-1-msj-10.1177_13524585231155639 – Supplemental material for Cerebrospinal fluid–related tissue damage in multiple sclerosis patients with iron rim lesions Click here for additional data file. Supplemental material, sj-doc-1-msj-10.1177_13524585231155639 for Cerebrospinal fluid–related tissue damage in multiple sclerosis patients with iron rim lesions by Matthias Wittayer, Claudia E Weber, Maximilian Kittel, Michael Platten, Lucas Schirmer, Hayrettin Tumani, Achim Gass and Philipp Eisele in Multiple Sclerosis Journal

sj-docx-2-msj-10.1177_13524585231155639 – Supplemental material for Cerebrospinal fluid–related tissue damage in multiple sclerosis patients with iron rim lesions Click here for additional data file. Supplemental material, sj-docx-2-msj-10.1177_13524585231155639 for Cerebrospinal fluid–related tissue damage in multiple sclerosis patients with iron rim lesions by Matthias Wittayer, Claudia E Weber, Maximilian Kittel, Michael Platten, Lucas Schirmer, Hayrettin Tumani, Achim Gass and Philipp Eisele in Multiple Sclerosis Journal
