# Supplementary material for: Cerebrospinal fluid–related tissue damage in multiple sclerosis patients with iron rim lesions
Source: Mult Scler. 2023 Feb 19;29(4-5):549–58. doi: 10.1177/13524585231155639 (PMC10152561; doi:10.1177/13524585231155639)
Supplement: sj-doc-1-msj-10.1177_13524585231155639 – Supplemental material for Cerebrospinal fluid–related tissue damage in multiple sclerosis patients with iron rim lesions [file sj-doc-1-msj-10.1177_13524585231155639.doc]

**MS Journal Appendix for MRI methodology**

| Hardware | |
| --- | --- |
| Field strength | 3 T |
| Manufacturer | Siemens Healthineers, Erlangen, Germany |
| Model | SKYRA |
| Coil type  (e.g. head, surface) | Head |
| Number of coil channels | 20-channel head coil |

| Acquisition sequences | |
| --- | --- |
| Type | **3D MPRAGE** |
| Voxel size | 0.9 × 0.9 × 0.9 mm |
| TR | 1900 ms |
| TE | 2.49 ms |
| TI | 900 ms |
|  | |
|  | **3D FLAIR** |
| Voxel size | 0.5 × 0.5 × 0.9 mm |
| TR | 5000 ms |
| TE | 398 ms |
| TI | 1800 ms |
|  | |
|  | **Diffusion-weighted imaging** |
| TR | 5300 ms |
| TE | 68 ms |
| ST | 4 mm |
|  | |
|  | **SWI** |
| TR | 27 ms |
| TE | 20 ms |
| ST | 1.5 mm |
| Voxel size | 0.9 × 0.9 × 1.5 mm |

| Image analysis methods and outputs | |
| --- | --- |
| ***Lesions*** | |
| Type  (e.g. Gd-enhancing, T2-hyperintense, T1-hypointense) | Iron rim lesions, non-Iron rim lesions |
| Analysis method | Lesion segmentation |
| Analysis software | Lesion Segmentation Toolbox |
| Output measure  (e.g. count or volume [ml]) | ADC values (x 10-3 mm2/^s^) |
| ***Tissue volumes*** | |
| Type  (e.g. whole brain, grey matter, white matter, spinal cord) | NAWM, NAGM, DGM |
| Analysis method | FSL tools |
| Analysis software | FSL |
| Output measure  (e.g. absolute tissue volume in ml, tissue volume as a fraction of intracranial volume, percentage change in tissue volumes) | mL |

**Other analysis details:**

To investigate voxel-wise CSF-related tissue damage in lesions and the NAWM, tissue segmentation was achieved using the segmentation algorithm of SPM12 (Version 7771, Functional Imaging Laboratory, Wellcome Centre for Human Neuroimaging, London, UK; https://www.fil.ion.ucl.ac.ukon) on MPRAGE images, resulting in a probability map for CSF. Lesion masks were subtracted from the white matter masks to produce a NAWM mask. Moreover, to minimize partial volume effects, we excluded the first 2 mm next to the inner and outer CSF spaces, as well as lesion voxels < 1.5 mm. The CSF probability map was then binarized using FSL (https:// fsl.fmrib.ox.ac.uk/fsl/fslwiki/FSL). This binary CSF-mask was then overlayed with a distance transformation map using ITK-SNAP (http://www.itksnap.org/). Nonzero voxels were converted to ASCII-values, resulting in a two-dimensional matrix of distance and ADC values for each voxel in the lesion and NAWM mask.
