# Supplementary material for: Cerebrospinal fluid–related tissue damage in multiple sclerosis patients with iron rim lesions
Source: Mult Scler. 2023 Feb 19;29(4-5):549–58. doi: 10.1177/13524585231155639 (PMC10152561; doi:10.1177/13524585231155639)
Supplement: sj-docx-2-msj-10.1177_13524585231155639 – Supplemental material for Cerebrospinal fluid–related tissue damage in multiple sclerosis patients with iron rim lesions [file sj-docx-2-msj-10.1177_13524585231155639.docx]

**Cerebrospinal fluid-related tissue damage in multiple sclerosis patients with iron rim lesions**

*Supplemental material*

Matthias Wittayer; Claudia E. Weber; Maximilian Kittel; Michael Platten; Lucas Schirmer; Hayrettin Tumani; Achim Gass; Philipp Eisele

**Table S1:** z-matrix for comparison-of-correlation coefficients (r) of linear correlations of ADC values and distance from CSF in MS patients with ≥ 1 IRL and MS patients without IRLs

| First r  Second r | IRLs in MS patients with ≥ 1 IRL | Non-IRLs in MS patients with ≥ 1 IRL | All Lesions in MS patients with ≥ 1 IRL | NAWM in MS patients with ≥ 1 IRL | All Lesions in MS patients without IRLs | NAWM in MS patients without IRLs |
| --- | --- | --- | --- | --- | --- | --- |
| IRLs in MS patients with ≥ 1 IRL | 0 | -0.77 | -1.02 | -11.29 | -4.42 | -8.48 |
| Non-IRLs in MS patients with ≥ 1 IRL | 0.77 | 0 | -0.57 | -31.37 | -7 | -22.98 |
| All Lesions in MS patients with ≥ 1 IRL | 1.02 | 0.57 | 0 | -32.17 | -6.66 | -23.36 |
| NAWM in MS patients with ≥ 1 IRL | 11.29 | 31.37 | 32.17 | 0 | 13.08 | 36.64 |
| All Lesions in MS patients without IRLs | 4.42 | 7 | 6.66 | -13.08 | 0 | -7.32 |
| NAWM in MS patients without IRLs | 8.48 | 22.98 | 23.36 | -36.64 | 7.32 | 0 |

Red numbers show statistically significant differences between linear correlations at p < 0.001.

**Abbreviations:** CSF = cerebrospinal fluid; IRL = iron rim lesion; MS = multiple sclerosis; NAWM = normal-appearing white matter

Fisher’s z-test was performed using the cocor-package for R ([Diedenhofen, B. & Musch, J. (2015). cocor: A Comprehensive Solution for the Statistical Comparison of Correlations. PLoS ONE, 10(4): e0121945. doi:10.1371/journal.pone.0121945](http://journals.plos.org/plosone/article?id=10.1371/journal.pone.0121945))

**Table S2:** z-matrix for comparison-of-correlations of linear correlations of ADC values and distance from CSF in MS patients with high IgG quotient and MS patients with low IgG quotient

| First r  Second r | Non-IRLs in MS patients with high IgG quotient | IRLs in MS patients with high IgG quotient | All lesions in MS patients with high IgG quotient | NAWM in MS patients with high IgG quotient | IRLs in MS patients with low IgG quotient | Non-IRLs in MS patients with low IgG quotient | All lesions in MS patients with low IgG quotient | NAWM in MS patients with low IgG quotient |
| --- | --- | --- | --- | --- | --- | --- | --- | --- |
| Non-IRLs in MS patients with high IgG quotient | 0 | 5.16 | 0.7 | -18.78 | -5.37 | -0.33 | -1.56 | -25.7 |
| IRLs in MS patients with high IgG quotient | -5.16 | 0 | -4.92 | -10.44 | -7.57 | -5.13 | -5.68 | -12.31 |
| All lesions in MS patients with high IgG quotient | -0.7 | 4.92 | 0 | -20.43 | -5.57 | -0.9 | -2.16 | -27.59 |
| NAWM in MS patients with high IgG quotient | 18.78 | 10.44 | 20.43 | 0 | -1.74 | 12.73 | 11.73 | -30.27 |
| IRLs in MS patients with low IgG quotient | 5.37 | 7.57 | 5.57 | 1.74 | 0 | 5.16 | 4.78 | 0.37 |
| Non-IRLs in MS patients with low IgG quotient | 0.33 | 5.13 | 0.9 | -12.73 | -5.16 | 0 | -1.04 | -17.56 |
| All lesions in MS patients with low IgG quotient | 1.56 | 5.68 | 2.16 | -11.73 | -4.78 | 1.04 | 0 | -16.75 |
| NAWM in MS patients with low IgG quotient | 25.7 | 12.31 | 27.59 | 30.27 | -0.37 | 17.56 | 16.75 | 0 |

Red numbers show statistically significant differences between linear correlations at p < 0.001, green numbers indicate statistically significant differences between linear correlations at p<0.05.

**Abbreviations:** CSF = cerebrospinal fluid; IRL = iron rim lesion; MS = multiple sclerosis; NAWM = normal-appearing white matter

Fisher’s z-test was performed using the cocor-package for R ([Diedenhofen, B. & Musch, J. (2015). cocor: A Comprehensive Solution for the Statistical Comparison of Correlations. PLoS ONE, 10(4): e0121945. doi:10.1371/journal.pone.0121945](http://journals.plos.org/plosone/article?id=10.1371/journal.pone.0121945))
